# Skeletal muscle in aged mice reveals extensive transformation of muscle gene expression

**DOI:** 10.1186/s12863-018-0660-5

**Published:** 2018-08-08

**Authors:** I-Hsuan Lin, Junn-Liang Chang, Kate Hua, Wan-Chen Huang, Ming-Ta Hsu, Yi-Fan Chen

**Affiliations:** 1 VYM Genome Research Center, National Yang-Ming University, Taipei, 112 Taiwan; 2 Institute of Biochemistry and Molecular Biology, National Yang-Ming University, Taipei, 112 Taiwan; 3 Department of Pathology & Laboratory Medicine, Taoyuan Armed Forces General Hospital, Taoyuan, 325 Taiwan; 4 The Ph.D. Program for Translational Medicine, College of Medical Science and Technology, Taipei Medical University, No.250, Wu-Hsing Street, Taipei, 11031 Taiwan

**Keywords:** Aging, Skeletal muscle, Cardiac-related genes, RNA sequencing analysis, Muscle fibers, Defects on differentiation

## Abstract

**Background:**

Aging leads to decreased skeletal muscle function in mammals and is associated with a progressive loss of muscle mass, quality and strength. Age-related muscle loss (sarcopenia) is an important health problem associated with the aged population.

**Results:**

We investigated the alteration of genome-wide transcription in mouse skeletal muscle tissue (rectus femoris muscle) during aging using a high-throughput sequencing technique. Analysis revealed significant transcriptional changes between skeletal muscles of mice at 3 (young group) and 24 (old group) months of age. Specifically, genes associated with energy metabolism, cell proliferation, muscle myosin isoforms, as well as immune functions were found to be altered. We observed several interesting gene expression changes in the elderly, many of which have not been reported before.

**Conclusions:**

Those data expand our understanding of the various compensatory mechanisms that can occur with age, and further will assist in the development of methods to prevent and attenuate adverse outcomes of aging.

**Electronic supplementary material:**

The online version of this article (10.1186/s12863-018-0660-5) contains supplementary material, which is available to authorized users.

## Background

Aging is a process whereby various changes were accumulated over time, resulting in dysfunction in molecules, cells, tissues and organs. The enormous variation in lifespan maintenance among different species points to genetic factors that specify an organism’s potential to reach old age. Specific genes implicated in the lifespan control have been identified in higher model organisms including worms [1], fruit flies [2], and mice [3]. Some protein products can trigger senescence in tissues or organs through the circulation system; others are potential biomarkers of aging that are released into blood circulation. For instance, Sirtuin 1 (SIRT1) declines at the transcriptional and translational levels in various tissues with age [4–6]. Activation and overexpression of SIRT1 reduces the oxidative stress and inflammation associated with ameliorating diseases, such as vascular endothelial disorders, neurodegenerative diseases, as well as skeletal muscle aging [7–10]. However, the aging phenotype is extremely complicated and there is currently no reliable and universal marker for aging.

Skeletal muscle is very important for movement and whole-body metabolisms regulated by various kinds of myokines. Skeletal muscle has been classified into several fiber types depending on the expression of myosin isoforms, twitch duration, shortening velocity, endurance, and metabolic mechanisms used for energy production. One of the major fiber types is type I fibers, also called slow twitch, red, or oxidative myofiber that have high amounts of myoglobin and mitochondria for oxidative metabolism. Another major fiber type is type II fibers, also known as fast-twitch, white, or glycolytic myofibers, which relies on glycolysis to generate energy due to less myoglobin and mitochondria. Type II fibers can be further categorized into type IIa (fast-twitch oxidative) and type IIb (fast-twitch glycolytic) fibers. During the aging process, the amount of type II fibers decreases, leading to a progressive decrease in the type II-to-type I fiber area ratio [11].

Sarcopenia, a term for the loss of skeletal muscle mass, strength, and function during the aging process [12], is a severe muscle disorder that impairs the quality of life in human society. Aging-induced sarcopenia has been characterized by the preferential loss of fast-twitch muscle fibers [13]. Many possible mechanisms have been reported in aged skeletal muscle, such as the increase of apoptosis, decrease in the number of satellite cells and regeneration rate, and dysfunction of autophagic and lysosomal degradation [14–16]. The detailed molecular mechanisms involved in the decline of skeletal muscle are still unclear. In this paper, we will present some interesting gene expression changes that occur with aging and most have not been reported before. We performed several phenotypic analyses to holistically explain the correlation between transcriptional changes, molecular mechanisms, and age-related phenotypes. Our findings can provide useful information for further research in the field of gerontology.

## Methods

### Animals

Wild-type (C57BL/6) male mice at 3 (young group) and 24 (old group) months of age were purchased from the National Laboratory Animal Center (Taipei, Taiwan) and maintained in a pathogen-free facility. 3-month-old mice was used as young age controls because by this time most of the tissues and organs were well-developed and the animals have reached sexual maturity. Euthanasia was performed using CO_2_ inhalation. At least four mice from each age group were sacrificed and the rectus femoris muscle was harvested from both legs of each mouse. The animal protocol was approved by the Institutional Animal Care and Use Committee of Taipei Medical University (LAC-2015-0297).

### RNA extraction and quantitative RT-PCR

Total RNA was isolated from mouse skeletal muscle (femoris muscle) using TRIzol Reagent (Life Technology). We execute real-time quantitative PCR using a TaqMan probe with TaqMan® Fast Universal PCR Master Mix and real-time PCR instrument (Thermo-Fisher Scientific). All amplifications were carried out in triplicate for each RNA sample and primer set. All measurements were done using RNA samples prepared from the rectus femoris muscles from four mice at young (3-month old) and old (24-month old) age. The amount of total input cDNA was calculated using hypoxanthine-guanine phosphoribosyltransferase as an internal control.

### Library preparation and sequencing

The sequencing library for mRNA-seq was prepared using TruSeq RNA Sample Preparation Kit v2 (Illumina) according to manufacturer’s instructions. Briefly, 4 μg of total high-quality RNA (RNA Integrity Number greater than 8) was subjected for poly-A mRNA isolation using poly-T oligonucleotides. The poly-A mRNA was fragmented and the first-strand cDNA was synthesized using random hexamers followed by second-strand cDNA synthesis, end repair, addition of a single A base and adapter ligation. The adapter-ligated double-stranded cDNA library was enriched with 7–9 cycles of PCR using KAPA HiFi DNA polymerase (Kapa Biosystems). Four RNA libraries were pooled together and sequenced on one lane of a HiSeq2500 flow cell (Illumina) by single-end sequencing with 100-bp read length to a depth of at least 30 million reads for each library. The RNA-seq data have been deposited in the ArrayExpress database under accession number E-MTAB-5176.

### Read mapping, quantification, and differential expression analysis

Quality control for the raw sequencing data was performed with FastQC v0.11.2 [17]. Reads were mapped to a database containing mouse rRNA and tRNA sequences using Bowtie2 v2.2.1 [18]. Subsequently, spliced alignment of unaligned reads to the mm9 assembly of the mouse genome (Illumina’s iGenomes) was performed with STAR v2.3.0e [19] and uniquely aligned reads were selected for downstream analysis. Read summarization was performed with featureCounts v1.4.6 [20] to obtain gene-level read counts over annotated genes (Gencode vM1). The level of gene expression was calculated as count-per-million using edgeR [21] in the R environment and lowly expressed genes were filtered. The difference in gene expression levels was calculated as the log2 fold changes of genes between young and old skeletal muscle samples.

### Functional profiling using gene ontology (GO)

In our RNA-seq dataset, there were 114 over-expressed and 634 under-expressed genes in 24-month old mice compared to the 3-month old mice. Top 100 differentially over-expressed and under-expressed genes which represent genes that were most dysregulated as the result of aging were selected for functional profiling. GO enrichment analysis was performed using the online tool provided by the Gene Ontology Consortium (http://geneontology.org/). Significant shared GO terms of the PANTHER GO-Slim molecular function, biological process and cellular component categories of the gene sets were reported (*P*-value < 0.05).

### Histopathology

Skeletal muscle (rectus femoris) was collected and fixed within 10% formalin buffered with phosphate, and subsequently embedded in paraffin. Tissue sections (4 μm) were subjected to hematoxylin-eosin (H&E) and immunohistochemistry (IHC) staining by standard procedures [22]. IHC staining was performed using paraffin-embedded skeletal muscle sections (4 μm). Sections were soaked in antigen retrieval buffer containing 10 mM sodium citrate (pH 9.0) and heated in a microwave. The sections were then incubated with primary antibody against Myh6 (Myosin heavy chain 6, 1:200, Proteintech) for 18–24 h at 4 °C, detected by biotinylated secondary antibodies and visualized by the LSAB Kit (DakoCytomation).

### Transmission electron microscopy (TEM)

Mouse skeletal muscle (rectus femoris) was fixed in a mixture of glutaraldehyde (1.5%) and paraformaldehyde (1.5%) in cacodylate buffer. They were post-fixed in 1% OsO_4_, and then rinsed in cacodylate buffer. Following dehydration, the tissues were embedded in Epon and sectioned for TEM as described previously [23]. Ultrastructure was observed using the Transmission Electron Microscope (Hitach HT-7700). We performed ultrastructural observation using 4 mice of each age (3-month and 24-month of age), 5 blocks of each mouse, and 3 grids of each block.

### Statistical analysis

Power analysis was performed with the *pwr* package in the R environment, and it indicates that 4 to 6 samples per group is required to achieve a power of 0.8 with effect size between 1.5 to 2.0 at a significance level of 0.05. All the results were presented as mean ± SD from four or more independent samples. Comparisons between two groups were calculated using 2-tailed heteroscedastic Student’s t test.

## Results

**Fig. 1:**
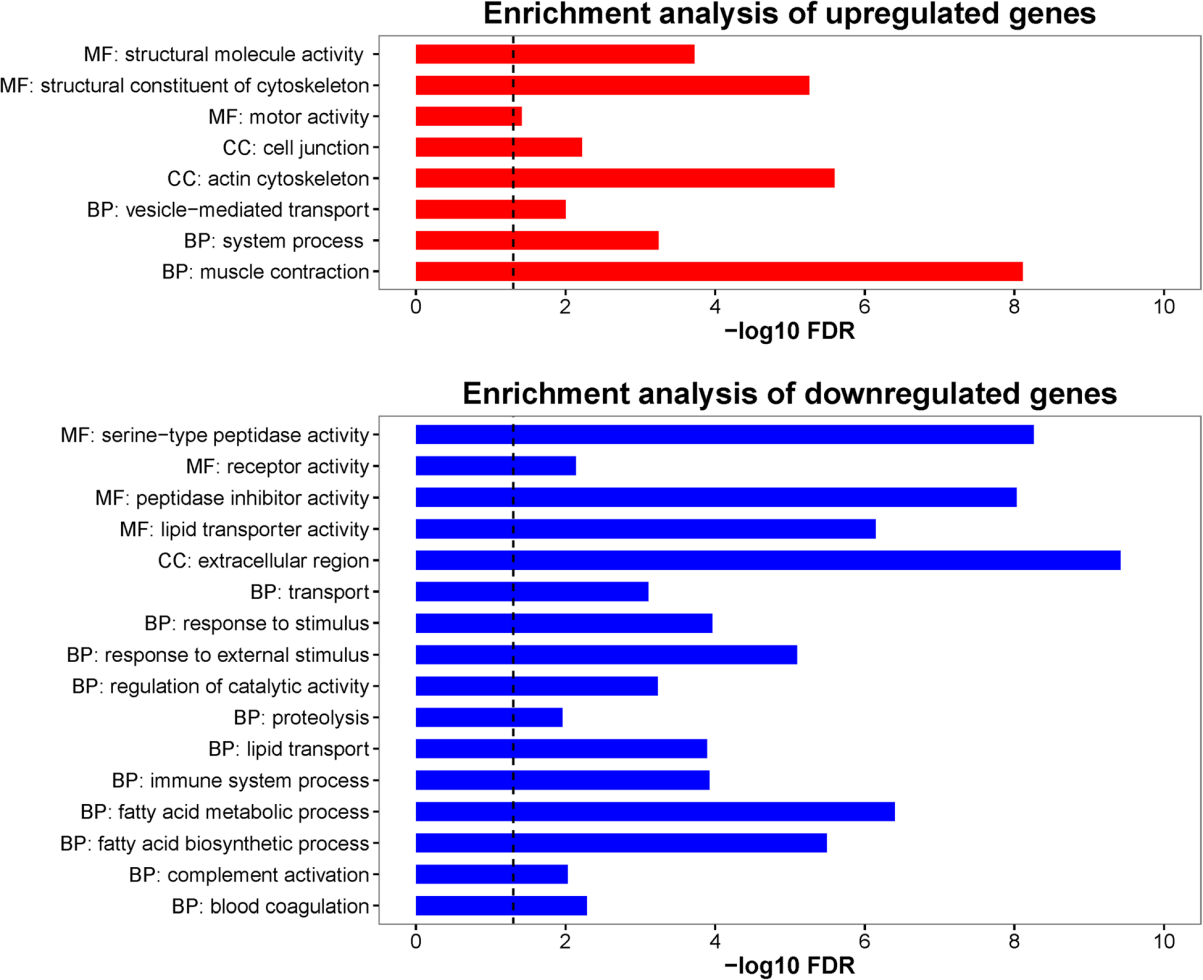
Gene expression fold changes and gene ontology enrichment analysis of age-related differentially expressed genes. Base on the fold changes of gene expression, the top 100 up-regulated and the top 100 down-regulated genes were carried out GO Enrichment Analysis (http://geneontology.org/page/go-enrichment-analysis). The three aspects of GO (biological processes, molecular functions, and cellular compartments) are abbreviated as BP, MF and CC respectively. The significance of enrichment is expressed as –log10 (false discovery rate). The black dashed line indicates the FDR-adjusted *P* value of 0.05

We compared the RNA levels between old (24-month-old) and young (3-month-old) skeletal muscle using high-throughput RNA sequencing analysis to evaluate the gene expression changes. Based on the fold changes in gene expression, we selected the top 100 up-regulated genes and 100 down-regulated genes for further analysis (Fig. 1). The results showed that the genes involved in muscle functions and metabolism were among the largest transcriptional changes observed in aged animals.

### Alterations in muscle sarcomere myosin gene expression: Up-regulation of embryonic and cardiac myosin genes in aging muscle

Transcriptome analysis revealed that some up-regulated genes were associated with muscle sarcomere structure (Additional file 1: Table S1). The embryonic form of myosins *Myh3* (Myosin heavy chain 3)*, Myh7* (Myosin heavy chain 7)*, Myl4* (Myosin light chain 4), and *Myl2* (Myosin light chain 2) as well as embryonic tropomyosin *Tnnt2* (Troponin T2, cardiac type) and embryonic heart gene *Ak147021* (Gm5532) were strongly up-regulated in 24-month-old muscle (Additional file 1: Table S1). Genes associated with type I muscle fibers, *Tnnt1* (Troponin T1, slow skeletal type) and *Atp2a2* (ATPase sarcoplasmic/endoplasmic reticulum Ca2+ transporting 2), were up-regulated in 24-month-old muscle (2.53- and 1.93- fold respectively), indicating a fast-to-slow muscle fiber transition during aging. Genes associated with both type I skeletal and cardiac muscles such as *Myl2*, *Myl3* (Myosin light chain 3), *Myoz2* (Myozenin 2)*,* and *Hspb7* (Heat shock protein family B (small) member 7) were up-regulated (2.79-, 2.19-, 1.65-, and 1.97-fold respectively). Additionally, sarcomere *Myom3* (Myomesin3) encoding a structural sarcomeric protein was found to be up-regulate (2.16-fold) in 24-month-old muscle.

**Fig. 2:**
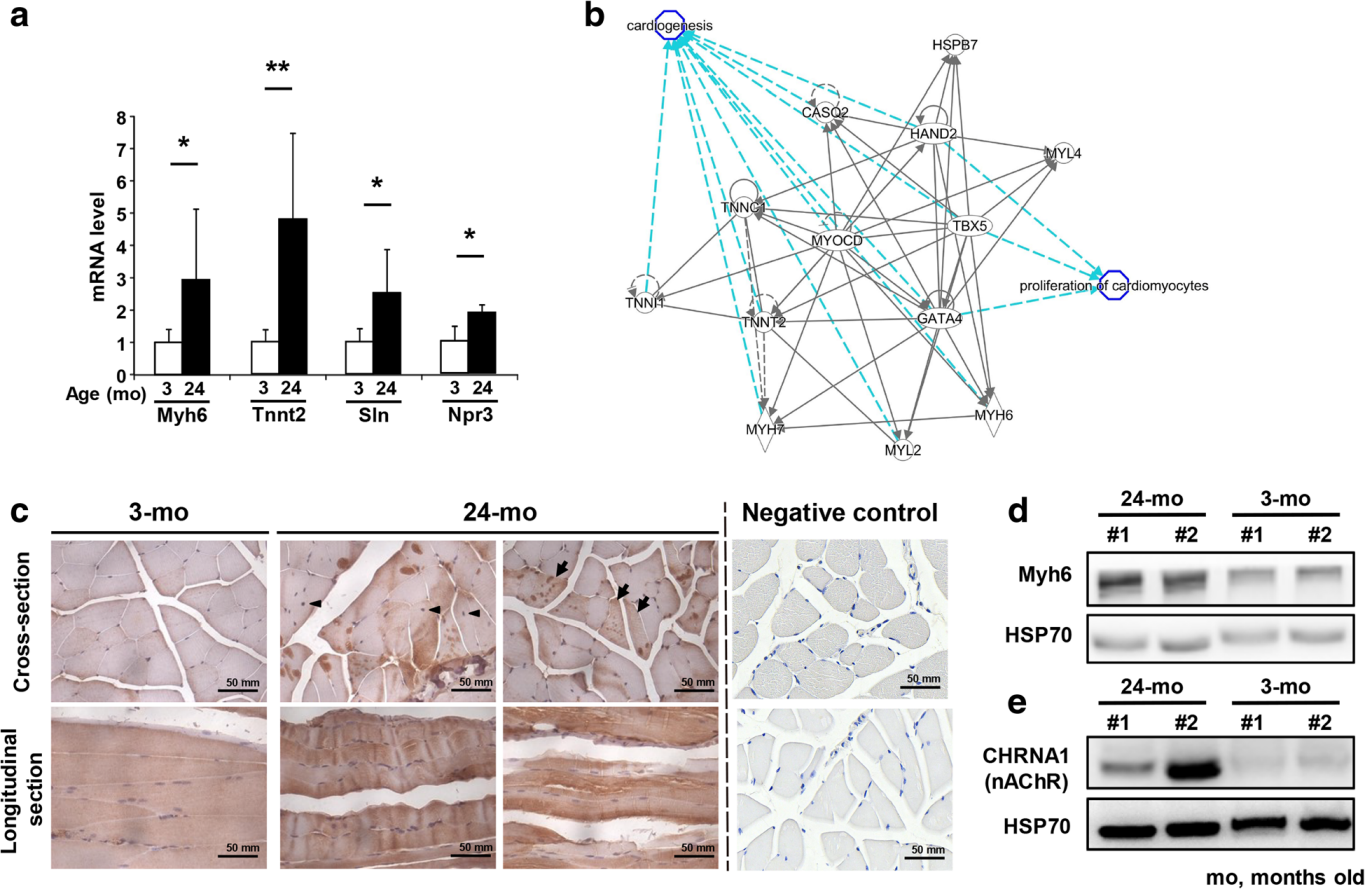
Age-related increased expression in skeletal muscle of aged mice compare to young mice. **a** Comparisons of the mRNA expression in skeletal muscle (rectus femoris) between wild-type old mice (24 months old) and wild-type young mice (3 months old). Four mice per age. The results are presented as the mean ± SD. ∗*p* < 0.05; ∗∗*p* < 0.005. **b** Increased expression of genes that are involved in cardiogensis and proliferation of cardiomyctes, such as *Gata4*, *Tbx5*, *Hand2,* and *Myocd*, as well as their downstream genes, *Tnnt2*, *Tnnc1*, *Myh6*, *Myh7*, *Casq2*, *Hspb7*, *Myl4*, *Myl2,* and *Tnni1*. These data were analyzed by using Ingenuity IPA software. **c** For the immunohistochemistry staining of Myh6, the brown signal indicates the Myh6 expressed positions. Arrow head indicate myofibers have undergone regenerative process. The black arrows indicate the degenerated myofibers. **d** Cardiac-specific myosin isoform Myh6 was detected by western blotting analysis. **e** The levels of nAChR (also known as CHRNA1) were detected in extracted proteins from 3- and 24-month-old rectus femoris. Heat shock protein 70 (HSP70) was used as an internal control. A total of 120 μg of protein from each fraction was loaded

We confirmed cardiac-related genes, including *Myh6*, *Tnnt2*, *Sln* (Sarcolipin)*,* and *Npr3* (Natriuretic peptide receptor 3), that were elevated in the aged skeletal muscle compared to young muscle (Fig. 2a). Ingenuity pathway analysis (IPA) showed that up-regulated genes were involved in cardiogenesis (Fig. 2b), such as *Gata4* (GATA binding protein 4), *Tbx5* (T-box 5), *Hand2* (Heart and neural crest derivatives expressed 2)*,* and *Myocd* (Myocardin), and in cardiac muscle contraction such as *Ryr2* (Ryanodine receptor 2), as well as their downstream genes, *Tnnt2*, *Tnnc1* (Troponin C), *Myh6*, *Myh7*, *Casq2* (Capsequestrin, cardiac muscle isoform*)*, *Hspb7*, and *Tnni1* (Troponin I1). The embryonic *Myl4* gene, known to interact with other cardiac-specific factors, also had a 2.48-fold increase in mRNA expression in the 24-month-old muscle (Fig. 2b, Additional file 1: Table S1). We evaluated the protein expression pattern of the cardiac-specific myosin isoform Myh6 in the skeletal muscle of young and old mice. Both the IHC staining and Western blotting analysis showed that aged skeletal muscle had higher Myh6 expression compared to young muscle (Fig. 2c-d). More stress accumulation and less repair capacity resulted in increased degenerated myofibers and regenerating myofibers (centrally located nuclei) observable in aged skeletal muscle; interestingly, Myh6 seems to have higher expression in the degenerated myofibers as indicated in Fig. 2c. We also observed other genes involved in heart function to have age-associated changes. *Pln* (Phospholamban), which is expressed mainly in heart that inhibits sarcoplasmic reticulum Ca^++^ATPase pumps and regulates myocardiac contractility, was highly up-regulated (8.84-fold). Gene encoding the transcriptional repressor Hdac9 (Histone deacetylase 9), which acts as a negative feedback regulator of myocyte differentiation, muscle endplate reinnervation, and cardiac hypertrophy, was up-regulated (2.60-fold) in the aged muscle. Transcription factor Mef2c (Myocyte enhancer factor 2C), which activates the expression of downstream cardiac genes during aging and repair/regeneration, was also transcriptionally up-regulated (1.25-fold). In addition, several genes involved in calcium ion metabolism were also up-regulated. For example, the cardiac-specific *Ryr2* gene for mediating the release of sarcoplasmic calcium ion, and *Trpc3* (Transient receptor potential cation channel, subfamily C, member 3), involved in forming calcium ion channels in both skeletal and cardiac muscles were also up-regulated in aged skeletal muscles (4.69- and 2.73-fold respectively).

### Changes in Z-band gene expression in aging muscle

The vertebrate striated muscle Z-band connects actin filaments of opposite polarity from adjacent sarcomeres and allows tension to be transmitted along a myofibril during contraction. *Igfn1* (Immunoglobulin-like and fibronectin type III domain containing 1), *Ky* (Kyphoscoliosis peptidase), and *Flnc* (Filamin-C) are genes encoding the Z-band-associated proteins that provide structural support for skeletal muscle. All three genes as well as *Des* (Desmin) were up-regulated (1.63-, 1.35-, 1.50-, and 1.35-fold respectively) in 24-month-old muscle whereas the major component of sarcomere, *Actn1* (alpha-actinin 1), was down-regulated (1.68-fold). The up-regulation of *Igfn1* (1.63-fold) down-regulated protein synthesis by binding to the translation elongation factor eEF1A, and furthermore induced down-regulation of muscle protein biosynthesis in the 24-month-old muscle. *Ky* is specifically expressed in skeletal and cardiac muscles, and thus the up-regulation of *Ky* and *Flnc* may indicate an alteration in Z-band for force generation in the aged muscle. In summary, the expression changes of genes involved in muscle function and differentiation in aged muscle suggest an overall diminishment in muscle contractile function during aging.

### Gene expression changes associated with the neuromuscular junction (NMJ)

Among the various factors involved in the development and function of the NMJ, we found that myotrophic and neurotrophic factor Igf1 (Insulin-like growth factor 1) was down-regulated (1.83-fold). Furthermore, several important genes associated with the NMJ, such as *ApoE* (Apolipoprotein E), *Dok7* (Docking protein 7), *Snta1* (Syntrophin alpha 1), and *Aqp4* (Aquaporin 4), as well as genes coding several serpins, exhibited a significant decrease in gene expression in aging muscle (Additional file 1: Table S1). This indicates the dysfunction of specific aspects of neuromuscular synaptogenesis during aging.

In contrast to the down-regulation of the NMJ genes described above, two of the genes encoding the acetylcholine receptor (AChR) subunits *Chrna1* (Cholinergic receptor nicotinic alpha 1 subunit) and *Chrnb1* (Cholinergic receptor nicotinic beta 1 subunit) as well as the co-receptor of agrin *Lrp4* (Low density lipoprotein receptor-related protein 4) were up-regulated (Additional file 1: Table S1, Fig. 2e). Lrp4 binds agrin released from motor neurons to regulate presynaptic differentiation at the NMJ. Lrp4 also binds with cholesterol carrier *ApoE*; hence, the up-regulation of *Lrp4* may serve to compensate the down-regulation of *ApoE* for the survival of neurons. It is also possible that the observed increase of *Chrna1*, *Chrnb1,* and *Lrp4* expressions reflect the change of muscle fiber types during aging as slow-twitch muscle fibers have wider NMJs and larger nerve terminal areas than the fast-twitch muscle fibers. We also observed strong up-regulation of the *Mib1* (Mindbomb E3 ubiquitin protein ligase 1) gene (3.33-fold), which plays a critical function in reducing the number of presynaptic boutons and branches at the NMJ. Thus, the interconnected transcriptional changes of genes associated with the NMJ during aging suggests a decreased functioning of the NMJ and a compensatory mechanism for the adaptation of changing muscle fiber types.

### Down-regulation of polyamine biosynthesis and amino acid metabolism: Implication for the loss of muscle mass during aging

**Fig. 3:**
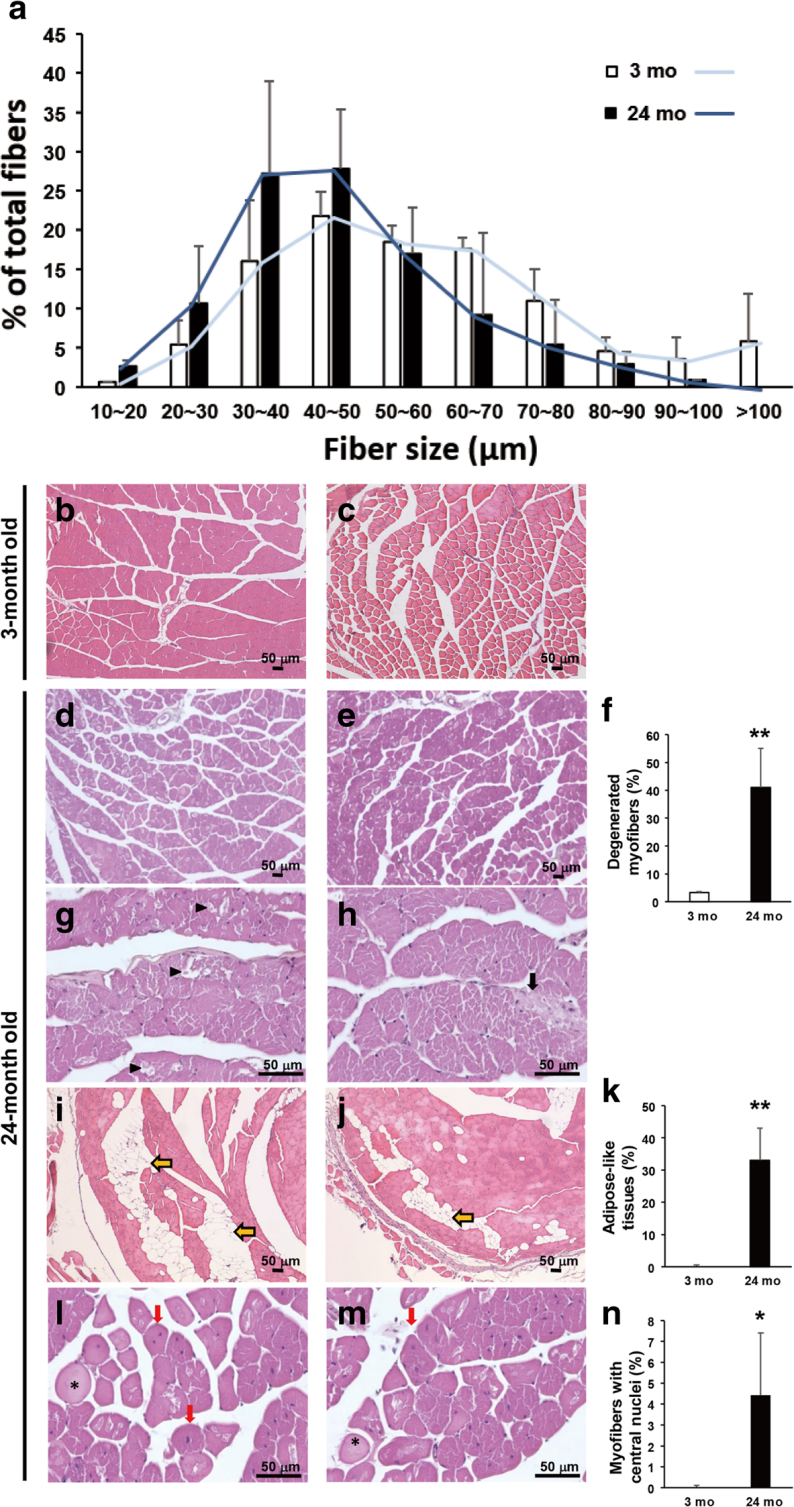
Increased adipose-like tissues, degeneration and regeneration of myofibers were observed in aged muscle. **a** Histogram of muscle fiber diameter sizing showed smaller fibers in aged mice. **b**-**c** Histological observation of rectus femoris obtained from 3-month-old wild-type mice. **d**-**e** Mild degeneration and severe lipid infiltration in the rectus femoris of 24-month-old mice. Yellow arrow indicates lipid infiltration. **f** Significant increase of the adipose-like tissues was found in aged mice. **g**-**j** The swelling fibers and severe degenerated fibers were observed in the skeletal muscle of 24-month-old mice. **k** Significant increase of the degenerated myofibers was observed in aged mice. **l**-**m** Myofibers with central nuclei, indicated regenerating myofibers, were found in the aged skeletal muscle. **n** Significant increase of the regenerated myofibers was found in aged mice. In (**f**), (**k**) and (**n**), there were 6 mice in each group; 6 micrographs (100X) for each mouse were conducted in a blinded fashion. The degenerated myofibers were defined and quantified follow the information on National Toxicology Program of NIH website. The area of adipose tissues was calculated using SPOT software. The results are presented as the mean ± SD. ∗*p* < 0.05; ∗∗*p* < 0.005. Tissues of rectus femoris were collected from male mice at 3 (young) and 24 (old) months of age. Black arrow indicates the collagen accumulation; black arrow head indicates the degenerated myofiber; red arrow indicates the regenerated myofiber; asterisk indicates the swollen myofiber

Muscle hypertrophy and mass are positively correlated with polyamine accumulation. We observed down-regulation of *Odc1* (Ornithine decarboxylase 1) and *Amd1* (Adenosylmethionine decarboxylase 1), both encoding key enzymes involved in polyamine biosynthesis (2.65- and 1.97-fold respectively), in 24-month-old muscle. Further, *Smox* (spermine oxidase) and GR (glucocorticoid receptor) target *Fkbp5* (FK506 binding protein 5)*,* which help to maintain muscle mass, were also decreased (4.73- and 4.16-fold respectively). Furthermore, serine/threonine-protein kinase, encoded by *Sgk1* (Serum/glucocorticoid regulated kinase 1), which is involved in the maintenance of muscle mass through the down-regulation of proteolysis and autophagy, was down-regulated (1.72-fold) in the aged muscle. These results are consistent with the loss of muscular mass of skeletal muscle fibers during aging. On the other hand, Forkhead transcription factors *Foxo6* (forkhead box O6) and *Foxo1* (forkhead box O1)*,* which are involved in the maintenance of muscle mass, were down-regulated (2.00- and 2.27-fold respectively) in 24-month-old muscle. The measurement of myofiber diameter showed smaller myofibers in aged femoris rectus muscle compared with young one (Fig. 3a) Accordingly, decreased expression of those genes, which play critical functions on polyamine biosynthesis and amino acid metabolism, suggests the loss of muscle mass in aged mice.

### Down-regulation of carbohydrate, amino acid metabolism, and mitochondrial gene expression in aging skeletal muscle: Implication in loss of strength in aging muscle

Transcriptome analysis revealed the expression of several metabolic genes that were strongly down-regulated in the 24-month-old muscle in comparison with the 3-month-old muscle (Additional file 1: Table S2). Skeletal muscle is a highly energy-dependent tissue. Our data revealed a reduced expression of genes involved in glucose metabolism in aged muscle (Additional file 1: Table S2). First, genes involved in glycogen biosynthesis and utilization were down-regulated in the skeletal muscle of 24-month-old mice as compared with 3-month-old mice. For example, the *Ppp1r3c* (Protein phosphatase 1 regulatory subunit 3C) gene, which encodes the regulatory subunit of protein phosphatase-1 that is involved in the activation of glycogen synthase, was strongly down-regulated (3.67-fold).

Several enzymes involved in gluconeogenesis were also down-regulated in aged muscle. *Pck1* (Phosphoenolpyruvate carboxykinase 1)*,* which encodes cytosolic phosphoenolpyruvate carboxykinase and serves as the key control element in gluconeogenesis, was strongly down-regulated (3.54-fold). *Gpt2* (Glutamic pyruvate transaminase (alanine aminotransferase) 2)*,* which encodes mitochondrial alanine transaminase and participates in gluconeogenesis, was also down-regulated (2.06-fold). In addition, *Pgk1* (Phosphoglycerate kinase 1)*,* which encodes phosphoglycerate kinase that participates in glycolysis, was down-regulated (1.48-fold). *Slc16a3* (Solute carrier family 16 member 3)*,* which encodes MCT4 (Monocarboxylate transporter 4) lactate transporter involved in anaerobic glycolysis energy production of fast-twitch muscle fibers, was down-regulated (1.82-fold). On the other hand, the glycolysis inhibitor Rrad (Ras related glycolysis inhibitor and calcium channel regulator), which inhibits glucose uptake, was up-regulated (3.59-fold) in 24-month-old skeletal muscle. The down-regulation of genes that function in glycogen metabolism and glycolysis suggests that the aging muscle will not be able to sustain muscle contractile activities.

Besides carbohydrates, amino acids oxidation can serve as an energy source for muscle by converting amino acid into glucose. We found that several enzymes involved in amino acid catabolism and the urea cycle were down-regulated or silenced in aging muscle (Additional file 1: Table S2). There seems to be no alternative source of energy to compensate for the lack of energy in the muscle cells of aging animals; however, more research on the metabolic state of skeletal muscle is necessary to determine this.

The mitochondrial translational activator encoded by *Mss51* (Mitochondrial Translational Activator, as known as *Zmynd17*) is a fast-twitch fiber gene and was strongly up-regulated (4.66-fold) in aged muscle. Nr1d1 (Nuclear receptor subfamily, group D, member 1), which is a transcription repressor that coordinates circadian rhythms with metabolism was up-regulated 3.5-fold in 24-month-old skeletal muscle. This transcription regulator has been shown to regulate skeletal muscle mitochondria biogenesis and autophagic regulation. *Glul* (Glutamate-Ammonia Ligase), which encodes mitochondrial glutamine synthetase, was under-expressed (2.71-fold). This enzyme is involved in the regulation of glycogen synthesis via the up-regulation of glycogen synthase. *Slc25a25* (Solute Carrier Family 25 Member 25), which encodes ATP-Mg^2+^/P_i_ inner mitochondrial membrane solute transporter and participates in muscle ATP production, was also strongly down-regulated (4.99-fold) in 24-month-old muscle. In addition, we also observed strong down-regulation of the *Fkbp5* gene (4.16-fold), which regulates metabolism during stress. These results suggest that aging muscle has reduced ATP production and mitochondria activity, and particularly, aging muscles are less well-adapted to stress.

It is interesting that all the *Mup* (Major urinary protein) genes were silenced in the aged muscle (Additional file 1: Table S2). The major function of *Mup* is chemical communication but recent studies have shown that one of the members, *Mup1*, is involved in the regulation of glucose and lipid metabolism and energy expenditure.

### Changes in expression of fatty acid metabolism during aging

Another energy source for muscle contraction is fatty acids. Transcriptome analysis also revealed changes in the lipid metabolism (Additional file 1: Table S2). The *Lpin1* (Lipin 1) gene, which encodes phosphatidic acid phosphohydrolase was found to be down-regulated (1.53-fold) in 24-month-old muscle. *Fabp1* (Fatty acid-binding protein 1) and *ApoB* (Apolipoprotein B), which are involved in the transport of long chain fatty acid and fat molecules respectively, were found silenced in 24-month-old muscle (Additional file 1: Table S2). This result is consistent with the accumulation of adipose-like tissues in the 24-month-old muscle (Fig. 3b-f). Several apolipoprotein genes, such as *Apoc3* (Apolipoprotein C3)*, Apoa5* (Apolipoprotein A5) *and Apoc2/Apoc4* (Apolipoprotein C2/ Apolipoprotein C4)*,* were also silenced in the aged muscle.

**Fig. 4:**
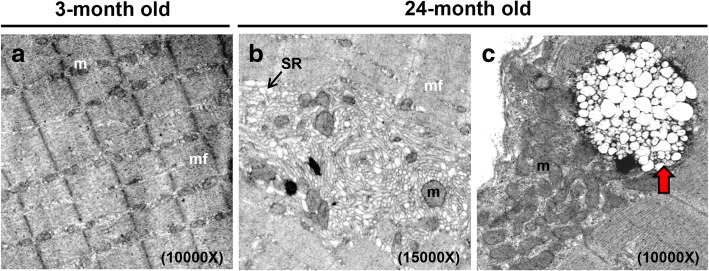
Degenerated myofilaments and mitochondrial breakdown in the muscle of aged mice. Ultrastructure of the rectus femoris from male mice (**a**) at the ages of 3 months and (**b**-**c**) 24 months old. **b** Aged rectus femoris muscle displayed large tubular aggregates, formed by SR. **c** The MVB was enlarged and shown in the aged skeletal muscle. The red arrow indicates the MVB. N, nucleus; m, mitochondria; mf, myofilament; SR, sarcoplasmic reticulum. We performed ultrastructural observation using 4 mice of each age, 5 blocks of each mouse, and 3 grids of each block. We screened each grid and collected more than 5 images for us to show the appropriate and reproducible data

Aside from adipose-like tissues infiltration, we also found severe degenerated myofibers accompanied by collagen accumulation (Fig. 3g-k) in the rectus femoris muscles of old mice. Based on the information from National Toxicology Program of NIH (https://ntp.niehs.nih.gov/nnl/musculoskeletal/skel_musc/degen/skeletal-muscle-degeneration_508.pdf), degenerated myofibers look either pale or dark; additionally histological observation includes various changes, such as cell swelling, vacuolation, loss of striation, fragmentation, and rupture of fibers. In aged skeletal muscle, we observed pale and swollen myofibers, ruptured fibers, as well as regenerated myofibers, centrally positioned nuclei in the myofibers (Fig. 3l-n). In Fig. 3f, k, n, we used blinded test to quantify degenerated myofibers, including pale, swelling, and ruptured fibers, meanwhile we also quantified myofibers that had centrally located nuclei that indicated myofibers under regeneration. Adipose tissues observed in skeletal muscle were measured using SPOT software to show the fat infiltration proportion. Subsequently, we analyzed the ultrastructure of rectus femoris muscle from young and aged mice. Transmission electron microscopy showed that myofilaments in young skeletal muscle were orderly and closely arranged (Fig. 4a); however, loose and twisted arrangements and enlarged sarcoplasmic reticulum (SR) were observed in aged muscle (Fig. 4b). The ultrastructure of aged skeletal muscle also showed enlarged multivesicular structure, which seems to be the late endosome or multivescular body (MVB) as well as apparent lipofuscin granules (Fig. 4c). Aged mice have a diminished capacity for the regeneration of skeletal muscle and displacement of differentiated adipose tissues, resulting in the degeneration and loss of skeletal myofibers.

### Changes in immune genes during aging

The switch from fast-to-slow muscle fiber was accompanied with a decrease in immune cell population and increase in cardiac vessel markers. In aged skeletal muscle, we found that specific genes related to macrophage, T lymphocytes, B lymphocytes, and complement system were silenced or strongly down-regulated (Additional file 1: Table S3) and vessel endothelial markers were up-regulated (Additional file 1: Table S4). For example, genes encoding the B-cell specific surface molecules *Cd19* (Cluster of differentiation 19)*, Cd79a* (Cluster of differentiation 79a) and *Cd22* (Cluster of differentiation 22) as well as *Btla* (B and T lymphocyte associated) and *Cxcr5* (Chemokine (C-X-C motif) receptor 5) of T cells and *Cd209b* (Cluster of differentiation 209b) of dendritic cells were silenced or strongly down-regulated in 24-month-old muscle (Additional file 1: Table S3). Angiogenesis and vasculogenesis in skeletal muscle have been shown to be modulated by monocytes and macrophages [24–27]. The decline of endothelial gene expression could lead to out-of-control blood pressure. Taken together, the aged skeletal muscle cells and their environment have reduced immune functions compared with young individuals. The repair/regeneration of muscle injury is compromised with the decline of immune function, which further accelerates the aging process.

## Discussion

Comparative transcriptome analysis between young and aged muscles revealed an extensive and complex transcriptional programming of genes associated with muscle structural proteins, NMJ, energy metabolism, stress response, polyamine biosynthesis, and immune functions. These changes probably reflect the adaptation of muscle to reduce energy metabolism and muscle loading during aging.

Muscle aging is associated with both the loss of muscle fibers and reduction in the mass and strength of muscle. Indeed, several type I muscle fiber genes were found to be up-regulated, and there is a switch to intermediate fast-twitch muscle fiber during aging, which employs both aerobic metabolism/respiration as well as glycolysis as an energy source. The lack of expression changes of type IIb muscle genes are contrary to the histological observation of a decrease in type II muscle in aging muscle. The discrepancy suggests a compensatory overexpression of these genes in the remaining type IIb fibers. Besides the up-regulation of type I slow-twitch muscle genes, we also observed the up-regulation of cardiac muscle genes and genes associated with both slow-twitch and cardiac muscles.

There are many structural and functional differences between cardiac and skeletal muscles, including the calcium source and mitochondria occupation. They also share some similar properties, such as having striated structures and performing aerobic metabolism (cardiac and type I skeletal muscle fibers). Recent studies have reported that proteins that are abundant in cardiac muscle are also detectable in skeletal muscle, such as construction-related proteins (*MYH7, MYL3,* and *TNNC1*) and calcium modulating proteins (*CASQ2*) [28]. Somehow, the expression of proteins with similar functions, which are originally expressed in cardiac fibers, was activated in skeletal muscle. We hypothesize that this is another compensatory mechanism for aged skeletal muscle. For example, recent reports have shown that the long non-coding RNA (*Mhrt*) and microRNA (miR-208b) were involved in regulating *Myh7* and *Myh6* expression in cardiac muscle [29, 30]. Therefore, when skeletal muscles regenerate with aging, increased *Myh7* expression may be accompanied by enhanced *Myh6* expression. In our studies, we speculated that the loss of type II fibers triggers cardiac myofibers formation to compensate the gradual loss of aged skeletal muscle. This result suggests that aging muscle reprograms its genes in response to low mechanical loading and repair/regeneration.

The up-regulation of embryonic forms of myosin heavy chains was observed in aging muscle. Because embryonic muscle has a low loading strength, the up-regulation of embryonic myosins is consistent with adaptation to reduced muscle loading of the aging muscle through functional compensation using different structural proteins. This is achieved through muscle regeneration during aging by the activation of muscle development process [31].

The innervation of muscle at the NMJ is not only required for muscular contraction but also for the maintenance of muscle tone and avoidance of muscle atrophy. A previous report suggests that NMJ dysfunction is associated with sarcopenia [32]. Our analysis showed genes associated with NMJ, such as *Igf1*, *ApoE*, *Dok7*, *Snta1,* and *Aqp4*, had altered expressions during aging. Injecting Igf1, a major metabolic regulator in muscle, has been shown to prevent aging-related motor neuron and NMJ degeneration [33]. *ApoE*-deficient (localized in the NMJ [34]) mice showed poor skeletal muscle healing during the chronic phase of reperfusion [35]. *Dok7* is essential for neuromuscular synaptogenesis through the activation of Muscle Associated Receptor Tyrosine Kinase (*MuSK*) [36]. Mice deficient in *Dok7* failed to form acetylcholine receptor clusters and neuromuscular synapses. *Snta1* binds to dystrophin and utrophin in muscle and plays an important role in synaptogenesis at the NMJ [37, 38]. *Snta1*-deficient muscles exhibit hypertrophy and aberrant formation of neuromuscular synapses deficient in utrophin [39, 40]. *Aqp4*, which is enriched in type II muscle fibers, has been shown to be associated with dystrophin and is lost or reduced in muscular dystrophy [41, 42]. Whether the changes in mitochondrial functions in muscle and nerve during aging affect the NMJ or whether the decline of the NMJ affects muscle functions and metabolism during aging remain to be elucidated. Our results suggest a potential compensatory mechanism during NMJ decline by up-regulating acetylcholine receptors.

We observed a strong down-regulation of genes participating in glucose metabolism including glycogen biosynthesis, gluconeogenesis, and glycolysis. The *Ppp1r3c* gene encodes the glycogen-targeting subunit of the protein phosphatase 1 (PP1) complex. In a previous study, the *Ppp1r3c* knockout mice resulted in enhanced insulin sensitivity and energy expenditure [43]. Pck1 also plays a role in metabolism in skeletal muscle. *Pck1* deficiency leads to loss of muscle tone and muscle weakness among other phenotypes [44].

Furthermore, the *Lpin1* and *Fabp1* genes involved in lipid metabolism were also strongly down-regulated. *Lpin1* is required for adipocyte differentiation and *Lpin1* deficiency has been shown to cause severe rhabdomyolysis in children [45]. *Mss51* is a skeletal muscle-specific gene and is highly expressed in muscles dominated by fast-twitch fibers. The genetic disruption of *Mss51* in myoblasts has been shown to result in increased levels of oxidative phosphorylation and ATP production as well as glycolysis and beta-oxidation [46]. *Mss51* forms a feedback translational regulatory loop in the production of mitochondrial Cox1 for mitochondrial cytochrome c oxidase (COX) assembly [47]; strong up-regulation of this gene can therefore result in the inhibition of mitochondrial energy metabolism. These results suggest that the energy metabolism of skeletal muscle becomes less effective during aging. How they are regulated, by internal and/or external signals, during the aging process is an interesting question that remains to be investigated.

Myofibers loss is one of the components of sarcopenia. It is mostly accompanied by inflammation, infiltration of adipose tissues, fibrosis, and decreased capillarogenesis. Several genes related to macrophage, T lymphocytes, B lymphocytes, and complement system were silenced or strongly down-regulated in aged skeletal muscles. For example, the transcription regulator, TSC22 domain family, member 3 encoded by *Tsc22d3*, which is anti-inflammatory and inhibits myogenic differentiation, was down-regulated in aged muscle.

Several genes, which participate in the pathological mechanisms for muscle disorders, had expression changes in aged skeletal muscle. The *Nrap* (Nebulin-related-anchoring protein) and *Xirp1* (Xin actin-binding repeat containing 1) genes, which are expressed in the myotendinous junction (MTJ) of skeletal muscle, were up-regulated (1.69- and 2.53-fold respectively) in aged muscle. *Xirp1* in particular has been found up-regulated in injured muscle and myopathy and both *Nrap* and *Xirp1* were associated with plaques in myofibrillar myopathies (MFMs) [48]. Batonnet-Pichon S. and colleagues had reported that six genes implicated MFMs, including *DES*, *FLNC*, *CRYAB* (Crystallin alpha B), *BAG3* (BCL2 associated athanogene 3), *ZASP/LDB3* (Z-band alternatively spliced PDZ motif-containing protein) and *MYOT* (myotilin) [49]. Intriguingly, DES, FLNC, CRYAB, BAG3 and ZASP/LDB3 were overexpressed in aged skeletal muscle. On top of that, microRNA miR-206, expressed specifically in skeletal muscle and shown to regulate skeletal muscle development, was down-regulated in the 24-month-old muscle tissue. Dysregulation of this microRNA is linked to human muscle degenerative diseases, such as amyotrophic lateral sclerosis (ALS) [50]. Summarized those finding, aging could be a big issue to acceleratediseases progress, and the similarity of the expression changes of critical genes between muscle diseases and aging implies some common mechanisms in their pathology and pathogenesis.

## Conclusions

Notable evidence in worms, fruit flies, mice, and humans suggest that skeletal muscle plays an important role in the systemic regulation of aging. In the future, potential biomarkers of aging through the endocrine system will be of clinical relevance and is a critical issue that needs our attention.

## Additional file

Additional file 1: **Table S1.** The expression fold changes of cardiac function-associated genes during aging process. **Table S2.** The expression fold changes of genes relating to metabolism during aging process. **Table S3.** The expression fold changes of immune-related genes during aging process. **Table S4.** The expression fold changes of genes relating to vessel functions during aging process. (DOCX 37 kb)

## Abbreviations

Actn1: alpha-actinin 1
ALS: amyotrophic lateral sclerosis
Amd1: Adenosylmethionine decarboxylase 1
Apoa5: Apolipoprotein A5
Apob: Apolipoprotein B
Apoc1: Apolipoprotein C1
Apoc2/Apoc4: Apolipoprotein C2/C4
Apoc3: Apolipoprotein C3
Apoe: Apolipoprotein E
Aqp4: Aquaporin 4
Atp2a2: ATPase sarcoplasmic/endoplasmic reticulum Ca2+ transporting 2
BAG3: BCL2 associated athanogene 3
Btla: B and T lymphocyte associated
Casq2: Capsequestrin, cardiac muscle isoform
Cd19: Cluster of differentiation 19
Cd209b: Cluster of differentiation 209b
Cd22: Cluster of differentiation 22
Cd79a: Cluster of differentiation 79a
Chrna1: Cholinergic receptor nicotinic alpha 1 subunit
Chrnb1: Cholinergic receptor nicotinic beta 1 subunit
COX: cytochrome c oxidase
CRYAB: Crystallin alpha B
Cxcr5: Chemokine (C-X-C motif) receptor 5
Des: Desmin
Dok7: Docking protein 7
Fabp1: Fatty acid-binding protein 1
Fkbp5: FK506 binding protein 5
Flnc: Filamin-C
Foxo1: Forkhead box O1
Foxo6: Forkhead box O6
Gata4: GATA binding protein 4
Glul: Glutamate-Ammonia Ligase
Gpt2: Glutamic pyruvate transaminase (alanine aminotransferase) 2
GR: Glucocorticoid receptor
H&E: Hematoxylin-eosin
Hand2: Heart and neural crest derivatives expressed 2
Hdac9: Histone deacetylase 9
Hspb7: Heat shock protein family B (small) member 7
Igf1: Insulin-like growth factor 1
Igfn1: Immunoglobulin-like and fibronectin type III domain containing 1
IHC: Immunohistochemistry
Ky: Kyphoscoliosis peptidase
Lpin1: Lipin 1
Lrp4: Low density lipoprotein receptor-related protein 4
MCT4: Monocarboxylate transporter 4
Mef2c: Myocyte enhancer factor 2C
MFMs: Myofibrillar myopathies
Mib1: Mindbomb E3 ubiquitin protein ligase 1
Mss51: Mitochondrial Translational Activator
MTJ: Myotendinous junction
Mup: Major urinary protein
MVB: Multivescular body
Myh: Myosin heavy chain
Myl: Myosin light chain
Myocd: Myocardin
Myom3: Myomesin3
MYOT: Myotilin
Myoz2: Myozenin 2
NMJ: Neuromuscular junction
Npr3: Natriuretic peptide receptor 3
Nr1d1: Nuclear receptor subfamily, group D, member 1
Nrap: Nebulin-related-anchoring protein
Odc1: Ornithine decarboxylase 1
Pck1: Phosphoenolpyruvate carboxykinase 1
Pgk1: Phosphoglycerate kinase 1
Pln: Phospholamban
Ppp1r3c: Protein phosphatase 1 regulatory subunit 3C
Rrad: Ras Related glycolysis inhibitor and calcium channel regulator
Ryr2: Ryanodine receptor 2
Sgk1: Serum/glucocorticoid regulated kinase 1
SIRT1: Sirtuin 1
Slc16a3: Solute carrier family 16 member 3
Slc25a25: Solute Carrier Family 25 Member 25
Sln: Sarcolipin
Smox: Spermine oxidase
Snta1: Syntrophin alpha 1
SR: Sarcoplasmic reticulum
Tbx5: T-box 5
TEM: Transmission electron microscopy
Tnnc1: Troponin C
Tnni1: Troponin I1
Tnnt1: Troponin T1, slow skeletal type
Tnnt2: Troponin T2, cardiac type
Trpc3: Transient receptor potential cation channel, subfamily C, member 3
Tsc22d3: TSC22 domain family, member 3
Xirp1: Xin actin-binding repeat containing 1
ZASP/LDB3: Z-band alternatively spliced PDZ motif-containing protein
