# Supplementary material for: Skeletal muscle in aged mice reveals extensive transformation of muscle gene expression
Source: BMC Genet. 2018 Aug 8;19:55. doi: 10.1186/s12863-018-0660-5 (PMC6083496; doi:10.1186/s12863-018-0660-5)
Supplement: Supplementary file 1 — Table S1. The expression fold changes of cardiac function-associated genes during aging process. Table S2. The expression fold changes of genes relating to metabolism during aging process. Table S3. The expression fold changes of immune-related genes during aging process. Table S4. The expression fold changes of genes relating to vessel functions during aging process. (DOCX 37 kb) [file 12863_2018_660_MOESM1_ESM.docx]

**Supplementary table 1. The expression fold changes of cardiac function-associated genes during aging process.**

| **Chr** | **Name** | **Abbreviation** | **SM_**  **3-mo** | **SM_**  **24-mo** | **Folds**  **(24-mo/3-mo)** |
| --- | --- | --- | --- | --- | --- |
| **Muscle morphology & contraction** | | | | | |
| chr2 | Myosin binding protein C, cardiac | **Mybpc3** | 0.24 | 4.87 | **20.29** |
| chr14 | Myosin heavy chain 6 | **Myh6** | 2.15 | 29.99 | **13.95** |
| chr1 | Troponin T2, cardiac type | **Tnnt2** | 2.15 | 20.83 | **9.69** |
| chr10 | Phospholamban | **Pln** | 2.02 | 17.86 | **8.84** |
| chr7 | Troponin I, cardiac 3 | **Dnaaf3/Tnni3** | 1.20 | 9.23 | **7.69** |
| chr11 | Myosin heavy chain 3 | **Myh3** | 1.91 | 7.12 | **3.73** |
| chr1 | Troponin I1 | **Tnni1** | 20.53 | 67.96 | **3.31** |
| chr5 | Myosin light chain 2 | **Myl2** | 81.60 | 227.60 | **2.79** |
| chr3 | Capsequestrin, cardiac muscle isoform | **Casq2** | 16.09 | 42.74 | **2.66** |
| chr14 | Myosin heavy chain 7 | **Myh7** | 115.20 | 293.20 | **2.55** |
| chr7 | Troponin T1, slow skeletal type | **Tnnt1** | 50.89 | 128.80 | **2.53** |
| chr14 | Troponin C | **Tnnc1** | 34.80 | 86.96 | **2.50** |
| chr11 | Myosin light chain 4 | **Myl4** | 5.88 | 14.61 | **2.48** |
| chr9 | Myosin light chain 3 | **Myl3** | 109.30 | 239.40 | **2.19** |
| chr4 | Myomesin3 | **Myom3** | 30.09 | 65.06 | **2.16** |
| chr2 | Myosin heavy chain 7b | **Myh7b** | 2.80 | 5.70 | **2.04** |
| chr4 | Heat shock protein family B (small) member 7 | **Hspb7** | 184.30 | 362.90 | **1.97** |
| chr2 | Fibrillin 1 | **Fbn1** | 37.23 | 60.32 | **1.62** |
| chr11 | Myosin heavy chain 10 | **Myh10** | 25.10 | 37.59 | **1.50** |
| **Calcium homeostasis (Responsible for ER calcium import and export)** | | | | | |
| chr10 | Phospholamban | **Pln** | 2.02 | 17.86 | **8.84** |
| chr9 | Sarcolipin | **Sln** | 1.71 | 8.98 | **5.25** |
| chr13 | Ryanodine receptor 2 | **Ryr2** | 0.75 | 3.52 | **4.69** |
| chr8 | Ras-related associated with diabetes | **Rrad** | 4.37 | 15.69 | **3.59** |
| chr3 | Transient receptor potential cation channel, subfamily C, member 3 | **Trpc3** | 2.56 | 6.98 | **2.73** |
| chr3 | Capsequestrin, cardiac muscle isoform | **Casq2** | 16.09 | 42.74 | **2.66** |
| chr5 | ATPase sarcoplasmic/endoplasmic reticulum Ca2+ transporting 2 | **Atp2a2** | 191.50 | 369.60 | **1.93** |
| chr3 | Myozenin 2 | **Myoz2** | 57.76 | 95.56 | **1.65** |
| chr6 | Inositol 1,4,5-trisphosphate receptor 1 | **Itpr1** | 60.56 | 99.09 | **1.64** |

**Supplementary table 1_continued. The expression fold changes of cardiac function-associated genes during aging process.**

| **Chr** | **Name** | **Abbreviation** | **SM_**  **3-mo** | **SM_**  **24-mo** | **Folds**  **(24-mo/3-mo)** |
| --- | --- | --- | --- | --- | --- |
| **Circadian rhythm** | | | | | |
| chr11 | Nuclear receptor subfamily, group D, member 1 | **Nr1d1** | 28.76 | 93.70 | **3.26** |
| chr6 | Basic helix-loop-helix family, member E40 | **Bhlhe40** | 70.84 | 132.20 | **1.87** |
| chr2 | Cryptochrome circadian clock 2 | **Cry2** | 41.98 | 63.74 | **1.52** |
| **Endothelial function** | | | | | |
| chr15 | Natriuretic peptide receptor 3 | **Npr3** | 16.77 | 53.07 | **3.16** |
| chr11 | Nitric oxide synthase 2 | **Nos2** | 1.71 | 4.11 | **2.40** |
| chr5 | Kinase insert domain receptor | **Kdr** | 35.62 | 58.32 | **1.64** |
| chr9 | Melanoma cell adhesion molecule | **Mcam** | 22.65 | 36.21 | **1.60** |
| chr11 | Plexin domain containing 1 | **Plxdc1** | 2.80 | 4.18 | **1.49** |
| chr15 | Platelet-derived growth factor receptors | **Pdgfb** | 17.56 | 24.91 | **1.42** |
| **Sarcomere** | |  |  |  |  |
| chr1 | Immunoglobulin-like and fibronectin type III domain containing 1 | **Igfn1** | 71.28 | 116.10 | **1.63** |
| chr6 | Filamin-C | **Flnc** | 552.70 | 827.00 | **1.50** |
| chr9 | Kyphoscoliosis peptidase | **Ky** | 200.90 | 270.90 | **1.35** |
| chr1 | Desmin | **Des** | 2382.00 | 3206.00 | **1.35** |
| chr12 | alpha-actinin 1 | **Actn1** | 17.39 | 10.36 | **0.60** |
| **Neuromuscular junction (NMJ)** | |  |  |  |  |
| chr18 | Mindbomb E3 ubiquitin protein ligase 1 | **Mib1** | 116.40 | 387.50 | **3.33** |
| chr2 | LDL receptor related protein 4 | **Lrp4** | 10.76 | 16.51 | **1.53** |
| chr11 | Cholinergic receptor nicotinic beta 1 subunit | **Chrnb1** | 29.65 | 43.53 | **1.47** |
| chr2 | Cholinergic receptor nicotinic alpha 1 subunit | **Chrna1** | 12.16 | 17.48 | **1.44** |
| chr5 | Docking protein 7 | **Dok7** | 22.85 | 16.07 | **0.70** |
| chr2 | Syntrophin alpha 1 | **Snta1** | 224.40 | 152.90 | **0.68** |
| chr10 | Insulin-like growth factor 1 | **Igf1** | 44.85 | 24.46 | **0.55** |
| chr7 | Apolipoprotein E | **ApoE** | 327.60 | 167.60 | **0.51** |
| chr18 | Aquaporin 4 | **Aqp4** | 58.47 | 27.95 | **0.48** |

**Supplementary table 1_continued. The expression fold changes of cardiac function-associated genes during aging process.**

| **Myotendon-muscle junction (MTJ)** | |  |  |  |  |
| --- | --- | --- | --- | --- | --- |
| chr9 | Xin actin-binding repeat containing 1 | **Xirp1** | 91.09 | 230.70 | **2.53** |
| chr19 | Nebulin-related-anchoring protein | **Nrap** | 961.90 | 1623.00 | **1.69** |
| chr6 | collagen, type I, alpha 2 | **Col1a2** | 355.20 | 126.80 | **0.37** |
| chr1 | collagen, type III, alpha 1 | **Col3a1** | 426.80 | 151.70 | **0.36** |
| chr11 | collagen, type I, alpha 1 | **Col1a1** | 189.40 | 63.95 | **0.34** |
| **Others** |  |  |  |  |  |
| chr12 | Histone deacetylase 9 | **Hdac9** | 3.45 | 8.98 | **2.60** |
| chr13 | Myocyte enhancer factor 2C | **Mef2c** | 797.20 | 1000.00 | **1.25** |

**chr, chromosome; SM_3-mo, skeletal muscle of 3-month-old mice; SM_24-mo, skeletal muscle of 24-month-old mice; logfc, log2 fold change.**

**Supplementary table 2. The expression fold changes of genes relating to metabolism during aging process.**

| **Chr** | **Name** | **Abbreviation** | **SM_**  **3-mo** | **SM_**  **24-mo** | **Folds**  **(24-mo/3-mo)** |
| --- | --- | --- | --- | --- | --- |
| **Carbohydrate metabolism** | | | | | |
| chr8 | Ras related glycolysis inhibitor and calcium channel regulator | **Rrad** | 4.37 | 15.69 | **3.59** |
| chrx | Phosphoglycerate kinase 1 | **Pgk1** | 1324.00 | 895.30 | **0.68** |
| chr11 | Solute carrier family 16 member 3 | **Slc16a3** | 410.20 | 225.90 | **0.55** |
| chr8 | Glutamic pyruvate transaminase (alanine aminotransferase) 2 | **Gpt2** | 498.10 | 241.90 | **0.49** |
| chr2 | Phosphoenolpyruvate carboxykinase 1 | **Pck1** | 139.50 | 39.46 | **0.28** |
| chr19 | Protein phosphatase 1 regulatory subunit 3C | **Ppp1r3c** | 1283.00 | 350.00 | **0.27** |
| chr13 | Fructose-1,6-biphosphatase 1 | **Fbp1** | 4.10 | 0.14 | **0.03** |
| chr4 | Aldolase B | **Aldob** | 18.20 | 0.03 | **0.00** |
| chr4 | Major urinary protein 7 | **Mup7** | 26.09 | 0.07 | **0.00** |
| chr4 | Major urinary protein 14 | **Mup14** | 8.74 | 0.03 | **0.00** |
| chr4 | α-1-microglobulin/bikunin precursor | **Ambp** | 10.21 | 0.00 | **Inf** |
| chr4 | Major urinary protein 1 | **Mup1** | 3.69 | 0.00 | **Inf** |
| chr4 | Major urinary protein 11 | **Mup11** | 15.13 | 0.00 | **Inf** |
| chr4 | Major urinary protein 17 | **Mup17** | 11.41 | 0.00 | **Inf** |
| chr4 | Major urinary protein 20 | **Mup20** | 86.35 | 0.00 | **Inf** |
| **Amino acid metabolism** | | | | | |
| chr10 | Serum/glucocorticoid regulated kinase 1 | **Sgk1** | 378.40 | 220.40 | **0.58** |
| chr10 | Adenosylmethionine decarboxylase 1 | **Amd1** | 2818.00 | 1434.00 | **0.51** |
| chr3 | forkhead box O1 | **Foxo1** | 51.10 | 25.50 | **0.50** |
| Chr4 | forkhead box O6 | **Foxo6** | 5.50 | 2.42 | **0.44** |
| chr12 | Ornithine decarboxylase 1 | **Odc1** | 512.00 | 192.90 | **0.38** |
| chr17 | FK506 binding protein 5 | **Fkbp5** | 143.50 | 34.51 | **0.25** |
| chr2 | Spermine oxidase | **Smox** | 855.60 | 180.80 | **0.21** |
| chr3 | Tryptophan 2,3-dioxygenase | **Tdo2** | 9.94 | 0.31 | **0.03** |
| chr13 | Betaine-homocysteine S-methyltransferase | **Bhmt** | 4.44 | 0.07 | **0.01** |
| chr8 | Tyrosine aminotransferase | **Tat** | 14.45 | 0.03 | **0.00** |
| chr14 | Methionine adenosyltransferase 1, alpha | **Mat1a** | 10.21 | 0.03 | **0.00** |
| chr5 | 4-hydroxyphenylpyruvic acid dioxygenase | **Hpd** | 5.70 | 0.00 | **Inf** |
| chr10 | Phenylalanine hydroxylase | **Pah** | 5.60 | 0.00 | **Inf** |
| chr6 | Camello-like 2 | **Cml2** | 3.72 | 0.00 | **Inf** |

**Supplementary table 2_continued. The expression fold changes of genes relating to metabolism during aging process.**

| **Chr** | **Name** | **Abbreviation** | **SM_**  **3-mo** | **SM_**  **24-mo** | **Folds**  **(24-mo/3-mo)** |
| --- | --- | --- | --- | --- | --- |
| **Lipid metabolism** | | | | | |
| chr2 | Low density lipoprotein receptor-related protein 4 | **Lrp4** | 10.76 | 16.51 | **1.53** |
| chr12 | Lipin 1 | **Lpin1** | 487.00 | 318.20 | **0.65** |
| chr7 | Apolipoprotein E | **Apoe** | 327.60 | 167.60 | **0.51** |
| chr7 | Apolipoprotein C1 | **Apoc1** | 16.50 | 1.73 | **0.10** |
| chr11 | Apolipoprotein H | **Apoh** | 7.00 | 0.31 | **0.04** |
| chr1 | Apolipoprotein A2 | **Apoa2** | 18.07 | 0.17 | **0.01** |
| chr12 | Apolipoprotein B | **Apob** | 24.66 | 0.03 | **0.00** |
| chr9 | Apolipoprotein A1 | **Apoa1** | 21.48 | 0.03 | **0.00** |
| chr6 | Fatty acid-binding protein 1 | **Fabp1** | 12.13 | 0.00 | **inf** |
| chr9 | Apolipoprotein C3 | **Apoc3** | 5.57 | 0.00 | **inf** |
| chr9 | Apolipoprotein A5 | **Apoa5** | 4.85 | 0.00 | **inf** |
| chr7 | Apolipoprotein C2/C4 | **Apoc2/Apoc4** | 3.25 | 0.00 | **inf** |
| **Xenobiotic metabolism** | | | | | |
| chr8 | Carboxylesterase 1f | **Ces1f** | 9.94 | 2.70 | **0.27** |
| chr7 | Cytochrome P450, family 2, subfamily E, polypeptide 1 | **Cyp2e1** | 241.00 | 42.84 | **0.18** |
| chr3 | Selenium binding protein 2 | **Selenbp2** | 4.95 | 0.28 | **0.06** |
| chr8 | Carboxylesterase 1C | **Ces1c** | 7.92 | 0.00 | **Inf** |
| chr5 | UDP glucuronosyltransferase 2 family, polypeptide B5 | **Ugt2b5** | 4.44 | 0.00 | **Inf** |
| chr5 | UDP glucuronosyltransferase 2 family, polypeptide B36 | **Ugt2b36** | 3.35 | 0.00 | **Inf** |
| **Mitochondrial functions** | | | | | |
| chr14 | Mitochondrial Translational Activator | **Mss51** | 49.73 | 231.80 | **4.66** |
| chr1 | Glutamate-Ammonia Ligase | **Glul** | 897.40 | 330.80 | **0.37** |
| chr2 | Solute Carrier Family 25 Member 25 | **Slc25a25** | 139.90 | 28.02 | **0.20** |

**chr, chromosome; SM_3-mo, skeletal muscle of 3-month-old mice; SM_24-mo, skeletal muscle of 24-month-old mice; logfc, log2 fold change.**

**Supplementary table 3. The expression fold changes of immune-related genes during aging process.**

| **Chr** | **Name** | **Abbreviation** | **SM_**  **3-mo** | **SM_**  **24-mo** | **Folds**  **(24-mo/3-mo)** |
| --- | --- | --- | --- | --- | --- |
| **Complement** | | | | | |
| chr1 | Complement component receptor 2 | **Cr2** | 9.22 | 0.07 | **0.01** |
| chr15 | Complement 9 | **C9** | 5.12 | 0.00 | **inf** |
| chr4 | Complement 8a | **C8a** | 4.47 | 0.00 | **inf** |
| chr2 | Complement C5 protein | **Hc** | 4.47 | 0.00 | **inf** |
| chr1 | Complement factor H-related 2 | **Cfhr2** | 3.11 | 0.00 | **inf** |
| **Macrophage** | | | | | |
| chr5 | FMS-like tyrosine kinase 1 | **Flt1** | 108.00 | 77.08 | **0.71** |
| chrX | Interleukin 13 receptor subunit alpha 1 | **Il13ra1** | 7.65 | 4.49 | **0.59** |
| chr11 | Cluster of differentiation 68 | **Cd68** | 6.35 | 3.28 | **0.52** |
| chr11 | Chemokine (C-C motif) ligand 8 | **Ccl8** | 13.76 | 2.32 | **0.17** |
| chr1 | Macrophage receptor | **Marco** | 21.79 | 0.03 | **0.00** |
| chr3 | CD5 antigen-like | **Cd5l** | 6.39 | 0.03 | **0.00** |
| **B lymphocyte** | | | | | |
| chr19 | Membrane-spanning 4-domains, subfamily A, member 6D | **Ms4a6d** | 4.44 | 1.38 | **0.31** |
| chr19 | Membrane-spanning 4-domains, subfamily A, member 6C | **Ms4a6c** | 10.90 | 2.83 | **0.26** |
| chr19 | Membrane-spanning 4-domains, subfamily A, member 6B | **Ms4a6b** | 14.93 | 3.08 | **0.21** |
| chr9 | Chemokine (C-X-C motif) receptor 5 | **Cxcr5** | 4.95 | 0.73 | **0.15** |
| chr19 | Membrane-spanning 4-domains, subfamily A, member 4C | **Ms4a4c** | 3.52 | 0.31 | **0.09** |
| chr4 | Cluster of differentiation 72 | **Cd72** | 4.58 | 0.35 | **0.08** |
| chr16 | B and T lymphocyte associated | **Btla** | 18.61 | 1.04 | **0.06** |
| chr12 | Immunoglobulin heavy constant gamma 2b | **Ighg2b** | 10.79 | 0.62 | **0.06** |
| chr19 | Membrane-spanning 4-domains, subfamily A, member 4B | **Ms4a4b** | 5.23 | 0.24 | **0.05** |
| chr12 | Immunoglobulin heavy constant gamma 1 | **Ighg1** | 3.21 | 0.17 | **0.05** |
| chr7 | Cluster of differentiation 79a | **Cd79a** | 15.17 | 0.66 | **0.04** |
| chr15 | Interleukin 7 receptor | **Il7r** | 6.83 | 0.28 | **0.04** |
| chr19 | Membrane-spanning 4-domains A1 | **Ms4a1** | 12.09 | 0.38 | **0.03** |
| chr11 | Cluster of differentiation 79b | **Cd79b** | 12.67 | 0.41 | **0.03** |
| chr16 | Immunoglobulin lambda constant 2 | **Iglc2** | 3.83 | 0.10 | **0.03** |
| chr7 | Cluster of differentiation 22 | **Cd22** | 17.39 | 0.31 | **0.02** |

**Supplementary table 3_continued. The expression fold changes of immune-related genes during aging process.**

| **Chr** | **Name** | **Abbreviation** | **SM_**  **3-mo** | **SM_**  **24-mo** | **Folds**  **(24-mo/3-mo)** |
| --- | --- | --- | --- | --- | --- |
| **B lymphocyte** | | | | | |
| chr7 | Cluster of differentiation 19 | **Cd19** | 10.28 | 0.17 | **0.02** |
| chr8 | Fc receptor, IgE, low affinity II, alpha polypeptide | **Fcer2a** | 9.32 | 0.17 | **0.02** |
| chr9 | POU domain, class 2, associating factor 1 | **Pou2af1** | 6.42 | 0.10 | **0.02** |
| chr7 | Spi-B transcription factor | **Spib** | 6.76 | 0.07 | **0.01** |
| **T lymphocyte** | | | | | |
| chr1 | Interleukin 1 receptor, type 1 | **Il1r1** | 16.87 | 11.02 | **0.65** |
| chr4 | Tumor necrosis factor receptor superfamily member 1B | **Tnfrsf1b** | 9.63 | 5.70 | **0.59** |
| chr2 | Integrin a4 | **Itga4** | 36.72 | 15.89 | **0.43** |
| chr10 | Integrin b2 | **Itgb2** | 10.96 | 4.60 | **0.42** |
| chr9 | Thymocyte antigen 1 | **Thy1** | 17.56 | 5.60 | **0.32** |
| chr14 | Lymphocyte cytosolic protein 1 | **Lcp1** | 39.31 | 10.26 | **0.26** |
| chr11 | Myosin 1G | **Myo1g** | 7.14 | 1.83 | **0.26** |
| chr3 | Cluster of differentiation 53 | **Cd53** | 15.44 | 3.35 | **0.22** |
| chr11 | Transcription factor 7 | **Tcf7** | 7.28 | 1.24 | **0.17** |
| chr9 | Chemokine (C-X-C motif) receptor 5 | **Cxcr5** | 4.95 | 0.73 | **0.15** |
| chr11 | T cell immunoglobulin and mucin domain containing 4 | **Timd4** | 7.99 | 1.00 | **0.13** |
| chr11 | Chemokine (C-C motif) ligand 5 | **Ccl5** | 3.04 | 0.31 | **0.10** |
| chr16 | B and T lymphocyte associated | **Btla** | 18.61 | 1.04 | **0.06** |
| chr2 | Cytohesin 1 interacting protein | **Cytip** | 9.29 | 0.59 | **0.06** |
| chr6 | Cluster of differentiation 4 | **Cd4** | 4.54 | 0.24 | **0.05** |
| chr15 | Interleukin 7 receptor | **Il7r** | 6.83 | 0.28 | **0.04** |
| chr4 | Lymphocyte protein tyrosine kinase | **Lck** | 4.27 | 0.17 | **0.04** |
| chr1 | Selection | **Sell** | 14.58 | 0.38 | **0.03** |
| chr17 | Lymphotoxin B | **Ltb** | 5.47 | 0.14 | **0.03** |
| chr6 | GTPase, IMAP family member 3 | **Gimap3** | 4.58 | 0.14 | **0.03** |
| chr11 | Chemokine (C-C motif) receptor 7 | **Ccr7** | 3.11 | 0.10 | **0.03** |
| chr6 | Cluster of differentiation 8a | **Cd8a** | 4.44 | 0.07 | **0.02** |

**Supplementary table 3_continued. The expression fold changes of immune-related genes during aging process.**

| **Chr** | **Name** | **Abbreviation** | **SM_**  **3-mo** | **SM_**  **24-mo** | **Folds**  **(24-mo/3-mo)** |
| --- | --- | --- | --- | --- | --- |
| **Inflammation** | | | | | |
| chr11 | WAP four-disulfide core domain 17 | **Wfdc17** | 4.82 | 2.11 | **0.44** |
| chr6 | Interferon regulatory factor 5 | **Irf5** | 12.94 | 4.56 | **0.35** |
| chr11 | Chemokine (C-C motif) ligand 8 | **Ccl8** | 13.76 | 2.32 | **0.17** |
| chr3 | S100 calcium binding protein A9 | **S100a9** | 3.89 | 0.28 | **0.07** |
| chr5 | Chemokine (C-X-C motif) ligand 13 | **Cxcl13** | 17.45 | 0.83 | **0.05** |
| chr1 | Macrophage receptor | **Marco** | 21.79 | 0.03 | **0.00** |
| chr3 | CD5 antigen-like | **Cd5l** | 6.39 | 0.03 | **0.00** |
| **Calcium regulation** | | | | | |
| chr11 | Tumor necrosis factor receptor superfamily member 13B | **Tnfrsf13b** | 4.44 | 1.00 | **0.23** |
| chr8 | Cluster of differentiation 209b | **Cd209b** | 25.92 | 3.77 | **0.15** |
| chr3 | S100 calcium binding protein A9 | **S100a9** | 3.89 | 0.28 | **0.07** |

**chr, chromosome; SM_3-mo, skeletal muscle of 3-month-old mice; SM_24-mo, skeletal muscle of 24-month-old mice; logfc, log2 fold change.**

**Supplementary table 4. The expression fold changes of genes relating to vessel functions during aging process.**

| **Chr** | **Name** | **Abbreviation** | **SM_**  **3-mo** | **SM_**  **24-mo** | **Folds**  **(24-mo/3-mo)** |
| --- | --- | --- | --- | --- | --- |
| **Blood endothelium** | | | | | |
| Chr10 | Basigin | **Bsg** | 515.50 | 411.40 | **0.80** |
| Chr14 | Stabilin 1 | **Stab1** | 30.33 | 22.87 | **0.75** |
| Chr19 | Cluster of differentiation 248 | **Cd248** | 15.47 | 11.54 | **0.75** |
| Chr5 | Fms-related tyrosin kinase 1 | **Flt1** | 108.00 | 77.08 | **0.71** |
| Chr2 | Protein C receptor | **Procr** | 14.99 | 9.78 | **0.65** |
| Chr11 | Angiotensin I converting enzyme | **Ace** | 84.47 | 53.52 | **0.63** |
| Chr14 | Integrin, beta-like 1 | **Itgbl1** | 15.44 | 8.22 | **0.53** |
| Chr9 | Intercellular adhesion molecule 1 | **Icam1** | 6.11 | 3.04 | **0.50** |
| Chr4 | Chemokine (C-C motif) ligand 21a | **Ccl21a** | 10.49 | 3.11 | **0.30** |
| Chr3 | Vascular cell adhesion molecule 1 | **Vcam1** | 23.94 | 6.05 | **0.25** |
| **Lymphatic endothelium** | | | | | |
| Chr11 | Fms-related tyrosin kinase 4 | **Flt4** | 9.73 | 7.53 | **0.77** |
| Chr4 | Podoplanin | **Pdpn** | 5.64 | 4.28 | **0.76** |
| Chr9 | Thymocyte antigen 1 | **Thy1** | 17.56 | 5.60 | **0.32** |
| Chr7 | Lymphatic vessel endothelial hyaluronan receptor 1 | **Lyve1** | 31.29 | 9.64 | **0.31** |
| Chr15 | Glycosylation dependent cell adhesion molecule 1 | **Glycam1** | 4.44 | 0.00 | **inf** |

**chr, chromosome; SM_3-mo, skeletal muscle of 3-month-old mice; SM_24-mo, skeletal muscle of 24-month-old mice; logfc, log2 fold change.**
